# Interprofessional communication (IPC) for medical students: a scoping review

**DOI:** 10.1186/s12909-020-02296-x

**Published:** 2020-10-16

**Authors:** Chermaine Bok, Cheng Han Ng, Jeffery Wei Heng Koh, Zhi Hao Ong, Haziratul Zakirah Binte Ghazali, Lorraine Hui En Tan, Yun Ting Ong, Clarissa Wei Shuen Cheong, Annelissa Mien Chew Chin, Stephen Mason, Lalit Kumar Radha Krishna

**Affiliations:** 1 Yong Loo Lin School of Medicine, National University of Singapore, 1E Kent Ridge Road, NUHS Tower Block, Level 11, Singapore, 119228 Singapore; 2 Division of Supportive and Palliative Care, National Cancer Centre Singapore, Level 4, 11 Hospital Drive, Singapore, 169610 Singapore; 3 Department of Pharmacy, Faculty of Science, National University of Singapore, Block S4A, Level 3, 18 Science Drive 4, Singapore, 117543 Singapore; 4 School of Life Science and Chemical Technology, Ngee Ann Polytechnic, 535 Clementi Road, Singapore, 599489 Singapore; 5 Medical Library, National University of Singapore Libraries, Centre for Translational Medicine, National University of Singapore, Blk MD6, 14 Medical Drive, #05-01, Singapore, 11759 Singapore; 6 Palliative Care Institute Liverpool, Academic Palliative & End of Life Care Centre, Cancer Research Centre, University of Liverpool, 200 London Road, Liverpool, L3 9TA UK; 7 Centre for Biomedical Ethics, National University of Singapore, Blk MD11, 10 Medical Drive, #02-03, Singapore, 117597 Singapore; 8 Duke-NUS Graduate Medical School, 8 College Road, Singapore, 169857 Singapore; 9 Division of Cancer Education, National Cancer Centre Singapore, Level 4, 11 Hospital Drive, Singapore, 169610 Singapore; 10 PalC, The Palliative Care Centre for Excellence in Research and Education, PalC c/o Dover Park Hospice, 10 Jalan Tan Tock Seng, Singapore, 308436 Singapore

**Keywords:** Interprofessional communication, Medical education, Undergraduate medical training, Medical students, Communications skills, Medicine

## Abstract

**Background:**

Effective Interprofessional Communication (IPC) between healthcare professionals enhances teamwork and improves patient care. Yet IPC training remains poorly structured in medical schools. To address this gap, a scoping review is proposed to study current IPC training approaches in medical schools.

**Methods:**

Krishna’s Systematic Evidence Based Approach (SEBA) was used to guide a scoping review of IPC training for medical students published between 1 January 2000 to 31 December 2018 in PubMed, ScienceDirect, JSTOR, Google Scholar, ERIC, Embase, Scopus and PsycINFO. The data accrued was independently analysed using thematic and content analysis to enhance the reproducibility and transparency of this SEBA guided review.

**Results:**

17,809 titles and abstracts were found, 250 full-text articles were reviewed and 73 full text articles were included. Directed Content analysis revealed 4 categories corresponding to the levels of the Miller’s Pyramid whilst thematic analysis revealed 5 themes including the indications, stages of trainings and evaluations, content, challenges and outcomes of IPC training. Many longitudinal programs were designed around the levels of Miller’s Pyramid.

**Conclusion:**

IPC training is a stage-wise, competency-based learning process that pivots on a learner-centric spiralled curriculum. Progress from one stage to the next requires attainment of the particular competencies within each stage of the training process. Whilst further studies into the dynamics of IPC interactions, assessment methods and structuring of these programs are required, we forward an evidenced based framework to guide design of future IPC programs.

## Background

Effective interprofessional communication (IPC) between healthcare professionals promotes teamwork, improves patient care and boosts cost efficiency [1, 2]. IPC also encourages open, honest and frank discussions, facilitates negotiations and resolution of conflicts, and promotes shared decision making [3]. These features foster coordinated medical, nursing, social, psychological and financial support by different members of the interprofessional team and contribute to holistic and longitudinal patient-centric care [4].

Yet, whilst medical schools have not been slow to recognise the importance of IPC training or equip its students to meet the IPC competencies set out by the Accreditation Council for Graduate Medical Education (ACGME) and the World Health Organisation’s Framework for Action on International Education & Collaborative Practice, significant diversity in the approaches and structuring of current IPC training in medical schools have been observed [5]. These variations create concern about the ability of medical students to function effectively in interprofessional teams upon graduation [6–8].

### The need for this review

To advance a consistent approach to IPC skills training in medical schools, a scoping review of current practice is proposed [9]. With most programs seen to be designed around the different levels of Miller’s pyramid this scoping review will frame it approach accordingly [6–8]. In addition, this scoping review will adopt a constructivist perspective and a relativist lens to capture IPC’s socioculturally-sensitive, linguistically-dependent, context and user-specific nature [10, 11] across different education and healthcare systems [12–16].

## Methods

A scoping review allows for the summarizing [17] of current approaches, pedagogies, assessments, and practice settings employed [18–20] in peer-reviewed and grey literature [12–16] and the circumnavigation of inevitable differences in practice, healthcare, education and healthcare financing across the different programs.

**Fig. 1:**
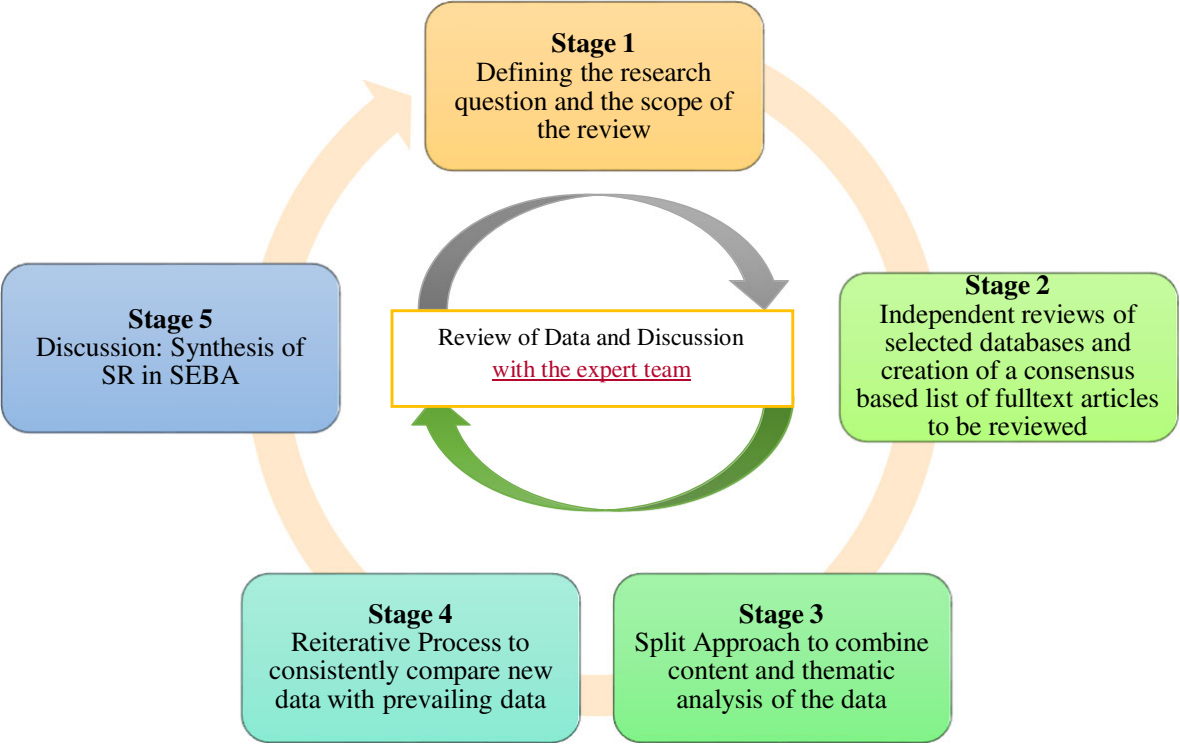
The SEBA Process

To guide this scoping review, we adopt Krishna’s Systematic Evidenced Based Approach [21, 22] (henceforth SEBA) to enhance transparency and reproducibility of the scoping review (Fig. 1). To begin SEBA employs an expert team comprising of local clinicians, educators, researchers, and a medical librarian to determine the research question and guide the scope of the review. SEBA structures its search process by adopting the approach used in systematic reviews. To enhance transparency of the review process SEBA uses trained researchers to carry independent searches for data across the selected databases including grey literature. These individual researchers use consensus based decisions to determine the final list of included articles. Independent reviews and consensus based determinations are also a part of SEBA’s ‘split approach’ which sees the concurrent use of thematic and content analysis of the data. The research team guided by the expert team review the findings and make comparisons of the findings with current available data as part of the reiterative process and the synthesis of the scoping review. SEBA also sees the employ of the PICO search strategy protocol and the Preferred Reporting Items for Systematic Reviews and Meta-Analyses Protocols (PRISMA-P) checklist [23]. SEBA also incorporates Levac et al. (2015) [24]‘s methodology for scoping reviews.

### Stage 1. Defining the research question and scope

Guided by the expert team, the research team identified the primary research question to be: “what are the characteristics of prevailing IPC programs?” The secondary research questions are: “what are the indications, training and evaluation methods, content, challenges and outcomes of these IPC programs?”

These questions were designed based on the population and concept elements of the inclusion and exclusion criteria, which are presented via a PICOS format in Table 1 [25]. For practical reasons, the ‘members’ of the IPC are drawn from a ‘small Multi-Disciplinary Team’ (MDT) which includes members from the faculties of medicine, nursing, physiotherapy, occupational therapy and social work [26]. Articles involving IPC training programs for medical students including healthcare professionals and or students from nursing, physiotherapy, occupational therapy and social work, were reviewed.

**Table 1:** PICOS, inclusion criteria and exclusion criteria applied to database search

PICOS	Inclusion Criteria	Exclusion Criteria
Population	• Undergraduate medical students from preclinical and clinical years • Nurses, and members of allied health including occupational therapist, physiotherapist and pharmacist, social workers	• Complementary medicine, non-medical specialties (e.g. Veterinary and Dentistry) and physicians outside of the Internal Medicine scope.
Intervention	• Pedagogy strategies in educating medical students about communication within the healthcare team through face-to-face or real-time virtual communication approaches	• Pedagogy strategies in educating other healthcare providers towards communicating with physicians
Comparison	• Comparison of pedagogy strategies, evaluation methods, outcomes and challenges in nurturing interprofessional communication	
Outcome	• Interprofessional communication strategies, evaluation methods on the effectiveness of educational practices • Outcomes and challenges in nurturing interprofessional communication	
Study design	• Articles in English or translated to English • All study designs including: ° Mixed methods research, randomized controlled trials, cohort studies, case-control studies, cross-sectional studies, and descriptive papers ° Case reports and series, ideas, editorials, and perspectives • Year of Publication: 1 January 2000–31 December 2018 • Databases: PubMed, Embase, CINAHL, Scopus, PsycINFO, ERIC, JSTOR, and Google Scholar	• Electronic and print information not controlled by commercial publishing

### Stage 2. Independent searches

Under the guidance of the expert team, search strategies (Supplementary File 1) were formulated with the following keywords: ‘medical students’, ‘nursing students’, ‘allied health students’, ‘interprofessional’, ‘communication’ and ‘education’. In keeping with Pham, Rajić [27]‘s approach to ensuring a viable and sustainable research process, the research team confined the searches to articles published between 1 January 2000 and 31 December 2018.

Seven trained researchers carried out independent searches of PubMed, Embase, CINAHL, Scopus, PsycINFO, ERIC, JSTOR, and Google Scholar databases and created independent lists of titles and abstracts to be scrutinized further based on the screening criteria as detailed in Table 1. The researchers discussed their findings at online meetings and determined the final list of full text articles to be reviewed using Sandelowski M [28]‘s ‘negotiated consensual validation’ approach.

#### Selection of studies for review

**Fig. 2:**
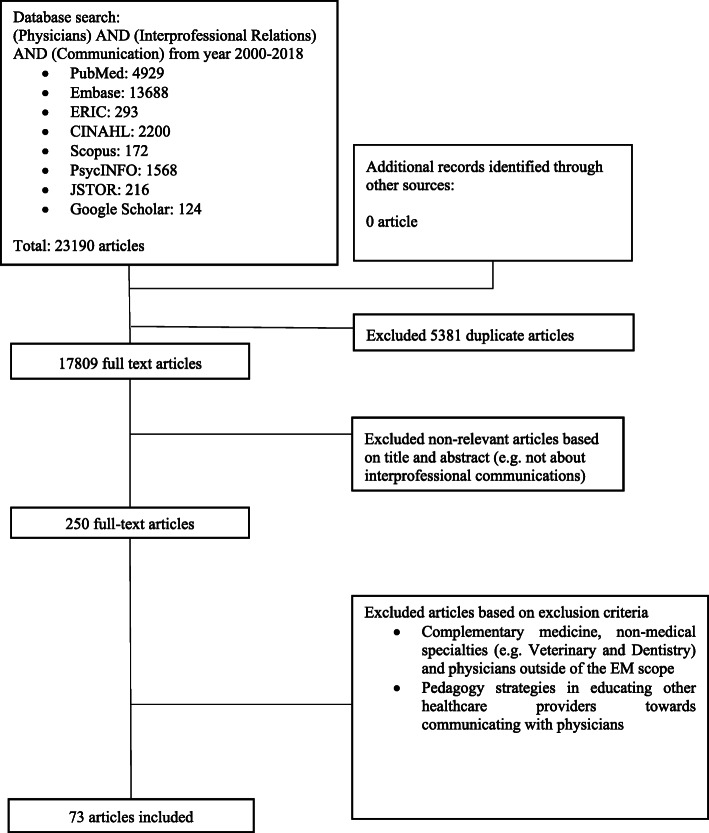
PRISMA Flowchart

The final list full text articles was independently scrutinised by members of the research team and discussed their findings at online meetings. The research team determined the final list of full text articles to be analysed using Sandelowski M [28]‘s ‘negotiated consensual validation’ approach. Figure 2 shows a summary of the PRISMA process.

### Stage 3. Data characterization and Split approach [21, 22, 29]

Inspired by the notion that communication skills training is a longitudinal process that develops in competency based stages, Hsieh and Shannon’s directed content analysis was adopted [30]. The codes and categories for this content analysis was drawn from various stages of the Miller’s Pyramid [6–8]. Miller’s Pyramid serves as an influential conceptual framework for the development and assessment of clinical competence, one which sees learners move from cognitive acquisition of knowledge to applied behaviour in clinical settings where beneficiaries reside. Critically an initial review of prevailing programs suggest that many IPC programs appear to fashion their programs around the 4 levels of Miller’s Pyramid [6–8] which are ‘Knows’ – which requires the learner to be aware of knowledge and skills, ‘Knows How’ – which sees the learner apply these knowledge and skills in theory, ‘Shows How’ – where knowledge and skills are applied in practice, and ‘Does’ – where the learner is shown to be able to function independently in the clinical setting [31].

The decision to adopt content analysis was not unanimous precipitating the employ of the ‘split approach’. The decision to adopt Braun and Clarke’s approach to thematic analysis [26] gained traction following the findings of the deductive category application. Part of the directed content analysis, the deductive category application suggested the presence of a number of other categories not related to the 4 levels of Miller’s Pyramid. These include the indications, structure, content, assessments and obstacles to IPC programs [14]. Omission of these critical categories and the belief that the adoption of predetermined categories based on Miller’s Pyramid required further evidencing, underpinned the decision to adopt Krishna’s ‘Split Approach’ [23–26].

The ‘Split Approach’ [29] sees two independent teams carry out concurrent reviews of the data using Hsieh and Shannon’s directed content analysis [30] and Braun and Clarke’s approach to thematic analysis [26]. This saw two members of the research team carry out concurrent and independent analyses of the data using Hsieh and Shannon’s directed content analysis [30] and three other members of the research team carry out simultaneous and independent analysis of the data using Braun and Clarke’s approach to thematic analysis [26]. The findings were discussed within each sub-team at online and face-to-face meetings where “negotiated consensual validation” was employed to determine the final list of themes and categories [32–34]. The themes from Braun and Clarke’s approach to thematic analysis [26] and the categories from Hsieh and Shannon’s directed content analysis [30] were compared [29].

### Stage 4. Review of results and comparing them with current data

Using PRISMA guidelines (Fig. 2), an initial search in eight databases revealed 17,809 titles and abstracts after removal of duplicates. Two hundred and fifty full-text articles were reviewed and a total of 73 articles were included for analysis. The narratives were written according to the Best Evidence Medical Education (BEME) Collaboration guide [35] and the STORIES (STructured apprOach to the Reporting In healthcare education of Evidence Synthesis) statement [36].

Scrutiny of the themes identified from the employ of Braun and Clarke’s approach to thematic analysis [26] and the categories identified from Hsieh and Shannon’s directed content analysis [30] were found to be overlap in some areas [29]. In addition the 5 themes identified using Braun and Clarke’s approach to thematic analysis [26] which were the indications, stages of trainings and evaluations, content, challenges and outcomes of IPC training were similar to the categories identified using Hsieh and Shannon’s directed content analysis [30]. This allowed the themes and categories to be presented together.

- Indications for IPC programs

The indications for the development of IPC programs are outlined in Table 2. Most accounts sought to assess perspectives towards Interprofessional work and communication, to introduce the use of IPC amongst medical students, to assess the nature of these interactions, determine roles and responsibilities of tutors and students in IPC, to better understand the process of problem solving and teamwork, to scrutinize the decision making processes that occurred in collaborations and evaluate the impact of debriefs and feedback sessions following IPC sessions. Many of these interactions took place in case discussions, simulations and or clinical practice and involved medical students in pre-clinical and clinical postings. Other accounts focused upon training faculty on teaching, facilitating IPC, setting and evaluating clinical competencies and debriefs and reports of IPC programs.

b. Stages of IPC training

**Table 2:** Indications for IPC Program

S/N	Title	Author	Objective of Study
1	Promoting Interprofessional Collaborative Practice Through Simulation	Alfes, C., et al	The purpose of this project was to educate faculty from the university’s schools of nursing, medicine, and physician assistant (PA) programs on the principles and best practices of simulation.
2	Design of a successful introductory interprofessional education experience	Helen, M. et al	This report describes an IPE mini- course for medical, nursing, and pharmacy students structured to meet the criteria of Allport’s Contact Hypothesis.
3	Developing Teamwork Using a Two-Tiered Debriefing Approach Clinical Simulation in Nursing	Andersen, P., et al	The study aims were as follows: 1. To determine students’ perceptions of interprofessional learning using immersive simulation. 2. To evaluate the impact of a two-tiered debriefing method on learning about teamwork during interprofessional immersive simulation, using a team performance observational tool (TPOT) during debriefing.
4	Interprofessional learning on polypharmacy	Anderson, E., et al	This short course enables final-year students to work with in-patients to meticulously assess the completeness and accuracy of their prescriptions.
5	Attitudes Toward Communication and Collaboration After Participation in a Mock Page Program: A Pilot of an Interprofessional Approach to Surgical Residency Preparation	J. Arumpanayil, A., et al	The purpose of this pilot study was to explore attitudes toward communication and collaboration among medical and nursing students, before and after participation in a mock page program.
6	Technology-enabled interprofessional education for nursing and medical students: A pilot study	Berg, B., et al.	This paper reports on a study that assessed the feasibility of conducting interprofessional SBAR training with nursing and medical students using remote technologies coupled with manikin simulation.
7	Evaluation of interprofessional education: lessons learned through the development and implementation of an interprofessional seminar on team communication for undergraduate health care students in Heidelberg - a project report	Berger, S., et al.	This project report describes the development, “piloting” and evaluation of an interprofessional seminar on team communication bringing together medical students and Interprofessional Health Care B.Sc. students at the Medical Faculty of Heidelberg University, Germany.
8	Mock pages are a valid construct for assessment of clinical decision making and interprofessional communication	Boehler, M. L., et al.	The purpose of this study is to assess standardized mock page cases as a valid construct to assess clinical decision making and interprofessional communication skills.
9	A mixed-methods study of interprofessional learning of resuscitation skills	Bradley, P., et al.	This study aimed to identify the effects of interprofessional resuscitation skills teaching on medical and nursing students’ attitudes, leadership, team-working and performance skills.
10	Examining the effects of interprofessional problem-based clinical ethics: Findings from a mixed methods study	Chihchen Chou, F., et al.	We aim to explore how IPE works in learning clinical ethics via PBL setting and how different professions’ perspectives influence each other via investigating the learning process and the students’ reflection.
11	The role of a multidisciplinary student team in the community management of chronic obstructive pulmonary disease	Clarke, H. and M. Voss	To determine whether a community-based, multidisciplinary team consisting of home-based caregivers and supervised students could improve the functional status and quality of life of patients living with chronic obstructive pulmonary disease (COPD) in a low-income, peri-urban setting in South Africa.
12	Fostering interprofessional communication through case discussions and simulated ward rounds in nursing and medical education: A pilot project	Wershofen, B., et al.	The aim of this project is to foster communication for medical and nursing students through interprofessional case discussions and simulated ward rounds as a form of training.
13	Learning to Overcome Hierarchical Pressures to Achieve Safer Patient Care: An Interprofessional Simulation for Nursing, Medical, and Physician Assistant Students	Reeves, S. A., et al.	This article describes an interprofessional simulation program to teach structured communication techniques to pre-professional nursing, medical, and physician assistant students.
14	An inter-professional approach to train and evaluate communication accuracy and completeness during the delivery of nurse-physician student handoffs	Maraccini, A., et al.	This study examined the impact of an interprofessional I-PASS training on communication accuracy and completeness during the delivery of nurse-physician student handoffs.
15	Interprofessional simulation-based education program: a promising approach for changing stereotypes and improving attitudes toward nurse-physician collaboration	Liaw, S. Y., et al.	The purpose of this study was to examine the effects of an interprofessional simulation-based communication education program on medical and nursing students’ perception on each other health profession and their attitudes toward nurse–physician collaboration.
16	Teamwork training with nursing and medical students: Does the method matter? Results of an interinstitutional, interdisciplinary collaboration	Hobgood, C., et al.	This study was designed and implemented to adapt the TeamSTEPPS content to pre-licensure nursing and medical students, and measure the effectiveness of four educational interventions at teaching this material.
17	An Interprofessional Workshop for Students to Improve Communication and Collaboration Skills in End-of-life Care	Erickson, J. M., et al.	Experiential interprofessional education workshops enhance perceptions about the benefits of teamwork, but further teaching and evaluation methods are needed to maximize the effectiveness.
18	COMPARISON of Communication Outcomes in Traditional VERSUS Simulation Strategies in Nursing and Medical Students	Reising, D. L., et al.	The purpose of this study is to understand interprofessional communication (nursing and medicine) within the context of the educational environment (traditional versus simulation).
19	The use of simulation and a modified TeamSTEPPS curriculum for medical and nursing student team training simulation	Robertson, B., et al.	We describe our adaptation of TeamSTEPPS for our curriculum and its use as an educational intervention for medical and nursing students.
20	“Collaborative-ready” students: Exploring factors that influence collaboration during a longitudinal interprofessional education practice experience	Rotz, M. and G. Dueñas	The objective of our study was to explore student-reported factors that influence collaboration within our longitudinal IPE experience
21	Medical school hotline: interprofessional education: future nurses and physicians learning together	Sakai, D. H., et al.	Implemented three collaborative learning experiences for first-year year medical and nursing students.
22	A novel interprofessional shadowing initiative for senior medical students	Shafran, D. M., et al.	This paper presents a unique opportunity to investigate the impact of a novel, multi-disciplinary and interprofessional educational experience for senior medical students.
23	The student-run free clinic: An ideal site to teach interprofessional education?	Sick, B., et al.	This article describes a prospective, observational cohort study of interprofessional attitudes and skills including communication and teamwork skills and attitudes about interprofessional learning, relationships and interactions of student volunteers in a SRFC compared to students who applied and were not accepted to the clinic and to students who never applied to the clinic.
24	Undergraduate students’ perceptions of and attitudes toward a simulation-based interprofessional curriculum: the KidSIM ATTITUDES questionnaire	Sigalet, E., et al.	The purpose of this present study was to examine the psychometric characteristics of the KidSIM Attitude Towards Teamwork in Training Undergoing Designed Educational Simulation (ATTITUDES) questionnaire.
25	Evaluation of an Interprofessional Education Communication Skills Initiative	Solomon, P. and J. Salfi	This study conducted a program evaluation of an innovative interprofessional communication skills initiative which incorporated problem-based learning, cooperative learning and standardized patients.
26	Undergraduate interprofessional education using high-fidelity paediatric simulation	Stewart, M., et al.	The aim of this study was to develop, implement and evaluate an interprofessional undergraduate programme using high-fidelity paediatric simulation to learn clinical competencies, and communication and teamworking skills.
27	An interprofessional approach to improving paediatric medication safety	Stewart, M., et al.	The aim of this study was to develop and evaluate an interprofessional teaching and learning workshop of paediatric dug prescribing and administration for medical and nursing students, which would facilitate learning of knowledge, core competencies, communication and team working skills. In addition, rigorous evaluation of the workshop could inform curriculum development.
28	Child disability case studies: an interprofessional learning opportunity for medical students and paediatric nursing students	Street, K. N., et al.	We describe an interprofessional learning [37] opportunity for pre-qualification medical and paediatric nursing students using community- based case studies of disabled children and their families.
29	Interprofessional simulation training improves knowledge and teamwork in nursing and medical students during internal medicine clerkship	M Tofil, N., et al.	We hypothesized that simulation training would improve both nursing students’ and medical students’ medical knowledge, communication skills, and understanding of each profession’s role in patient care.
30	Interprofessional training enhances collaboration between nursing and medical students: A pilot study	Turrentine, B., et al.	Interprofessional education is foundational to ensuring that students are prepared to engage in optimal collaboration once they enter clinical practice particularly in the care of complex geriatric patients undergoing surgery.
31	Analysis of an interprofessional home visit assignment: student perceptions of team-based care, home visits, and medication-related problems	Vaughn, L. M., et al.	The objective of this study was to determine the impact of an interprofessional medicine-pharmacy student home visit experience on students’ self-assessments of skills and abilities related to team-based care and identification of medication-related problems.
32	Developing interprofessional communication skills	Wagner, J., et al.	This article will describe the development and implementation of a pilot educational teaching/learning simulation exercise designed to promote teamwork and collaboration between medical students and nursing students.
33	Reflections and unprompted observations by healthcare students of an interprofessional shadowing visit	Wright, A., et al.	This paper reports work from a Centre for Interprofessional Practice in a higher education institution in the UK that offers four levels of interprofessional learning [37] to all healthcare students.
34	Interprofessional education: The student perspective	Lumague, M., et al.	In an effort to increase interprofessional collaboration, improve communication skills, foster respect and enhance knowledge of the different roles each discipline plays on the health care team, these students met together over a five week period and participated in interprofessional group sessions led by different health care professional leaders from the unit.
35	An introductory interprofessional exercise for healthcare students	Macdonnell, C., et al.	This workshop enabled the students to develop a better understanding of the approaches various health professionals use when caring for patients.
36	An Interprofessional Curriculum on Antimicrobial Stewardship Improves Knowledge and Attitudes Toward Appropriate Antimicrobial Use and Collaboration	MacDougall, C., et al.	A curriculum on antimicrobial stewardship consisting of independent learning and an interprofessional workshop significantly increased knowledge and attitudes towards collaborative antimicrobial stewardship among preclinical medical and pharmacy students.
37	An interprofessional education pilot program in maternity care: Findings from an exploratory case study of undergraduate students	Meffe, F., et al.	We hypothesized that the provision of this learning opportunity at the pre-licensure level would have the potential to diminish stereotypical thinking about other professional groups, increase awareness of others’ roles, responsibilities and scope of practice, and impact positively on one’s willingness to practice collaboratively with others.
38	Innovation in learning - An inter-professional approach to improving communication	Mitchell, M., et al.	This pilot project was founded on practice-based learning materials formulated specifically for undergraduate nursing and medical students in the setting of inter-professional small group tutorial.
39	Sustained effects of interprofessional shared learning on student attitudes to communication and team working depend on shared learning opportunities on clinical placement as well as in the classroom	Morison, S. and J. Jenkins	This study compares the attitudes, 1 year after experience of an undergraduate SL programme, of students who had participated in the programme with their peers who had not.
40	An interprofessional communication skills lab: A pilot project	Salvatori, P., et al.	The new IPE curriculum at McMaster is based on pedagogical principles of competency-based education and small group, problem-based learning
41	Evaluation of a Unique Interprofessional Education Program Involving Medical and Pharmacy Students	J. Nagge, J., et al.	This study was designed to evaluate self-reported changes in these domains using a validated pre-post survey instrument.
42	Integrating Collaborative Interprofessional Simulation into Pre-Licensure Health Care Programs	New, S. N., et al.	The team conference and acute care simulations provided students opportunities to practice interprofessional communication at various levels of care.
43	Students’ Perceptions on an Interprofessional Ward Round Training - A Qualitative Pilot Study	Nikendei, C., et al.	The present study aimed to analyse final year students’, nurses’ as well as physio- therapists’ views on a simulation-based interprofessional ward round training.
44	How can student experience enhance the development of a model of interprofessional clinical skills education in the practice placement setting?	O’Carroll, V., et al.	Exploring the student experience has assisted in developing relevant and accessible interprofessional learning opportunities within the practice placement setting.
45	Students’ understanding of teamwork and professional roles after interprofessional simulation-a qualitative analysis	Oxelmark, L., et al.	The present study investigates these concerns in a qualitative analysis of focus group data with undergraduate nursing and medical students after participating in IPSE.
46	Medical students’ engagement in collaborative communication during an interprofessional standardized patient encounter	K. Oza, S., et al.	Develop and apply a conceptual framework of ICC.
47	Development and implementation of an interprofessional pharmacotherapy learning experience during an advanced pharmacy practice rotation in primary care	Patel, K., et al.	The developed IPE program includes the use of case studies and problem-based learning methods to facilitate learning of conditions common in primary care as tools to improve interactions with health professions students.
48	A mile in their shoes: interdisciplinary education at the Johns Hopkins University School of Medicine	Pathak, S., et al.	The specific aim of this article is to describe our experience with creating an interdisciplinary elective for third- and fourth-year medical students at the Johns Hopkins University School of Medicine
49	Interprofessional training in the context of clinical practice: goals and students’ perceptions on clinical education wards	Ponzer, S., et al.	This paper describes the context of interprofessional training on clinical education wards (CEWs) and reports students’ perceptions of this type of interprofessional and professional training.
50	Evaluating an undergraduate interprofessional simulation-based educational module: communication, teamwork, and confidence performing cardiac resuscitation skills	Luctkar-Flude, M., et al.	The purpose of this study is to evaluate an innovative simulation-based IP educational module for undergraduate nursing and medical students on cardiac resuscitation skills.
51	Improving collaboration among medical, nursing and respiratory therapy students through interprofessional simulation	Elizabeth Ann King, A., et al.	Our study suggests that simulated scenarios can help interprofessional collaboration.
52	Interprofessional education for the quality use of medicines: Designing authentic multimedia learning resources	Levett-Jones, T., et al.	This paper describes the development of authentic multimedia resources that allow for participative, inter- active and engaging learning experiences based upon sound pedagogical principles.
53	What and how do students learn in an interprofessional student-run clinic? An educational framework for team-based care	Lie, D., et al.	To derive a framework for understanding student learning during team-based care provided in an interprofessional SRC serving underserved patients.
54	The impact of an interprofessional problem-based learning curriculum of clinical ethics on medical and nursing students’ attitudes and ability of interprofessional collaboration: A pilot study	Lin, Y.-C., et al.	Therefore, we conducted a pilot curricular study to evaluate the curricular impact on students’ confidence and attitude of interprofessional collaborative teamwork.
55	Interprofessional learning through shadowing: Insights and lessons learned	V. Kusnoor, A. and L. A. Stelljes	This study evaluates (1) how pre-clinical medical students describe the roles of the healthcare professionals they shadowed, and (2) whether shadowing can be used to introduce medical students to the benefits of interprofessional collaboration, and if so, in what ways.
56	Interprofessional student-led clinics: An innovative approach to the support of older people in the community	Kent, F., et al.	Undergraduate students, working in mixed professional teams, are able to deliver a useful additional health promotion service to older people.
57	Interprofessional clinical training for undergraduate students in an emergency department setting	Ericson, A., et al.	We conclude that training at an emergency department can provide excellent opportunities for interprofessional team training for undergraduate students.
58	Indonesian students’ participation in an interprofessional learning workshop	Ernawati, D., et al.	This study found that learning with other health students through an IPE workshop improved medical, nursing and pharmacy students’ attitudes towards the importance of shared learning, teamwork and communication in healthcare service.
59	Nursing and medical students teaming up: Results of an interprofessional project	Feather, R., et al.	
60	Simulating a patient’s fall as a means to improve routine communication: Joint training for nursing and fifth-year medical students	Flentje, M., et al.	To improve interprofessional communication and task management, a simulation-based emergency training session for nursing students and fifth-year medical students was developed at the KRH Klinikum Nordstadt in Hanover, Germany.
61	Effects of interprofessional education on patient perceived quality of care	Hallin, K., et al.	To assess the patients’ perceptions of collaborative and communicative aspects of care when treated by interprofessional student teams as compared to usual care.
62	Active interprofessional education in a patient based setting increases perceived collaborative and professional competence	Hallin, K., et al.	To evaluate whether students perceived that they had achieved interprofessional competence after participating in clinical teamwork training.
63	Interprofessional working in acute care	Holland, C., et al.	This paper describes the development and implementation of an interprofessional (IP) module for pre-qualification medical, nursing and physiotherapy students.
64	Development of an interprofessional educational module on infection control using high-fidelity patient simulation	Luctkar-Flude, M., et al.	This mixed methods study evaluated an interprofessional education infection control module as part of a larger action research project aimed at developing interprofessional health education using simulation
65	Developing a Foundation for Interprofessional Education Within Nursing and Medical Curricula	Leann Horsley, T., et al.	This article describes how a nursing and a medical school collaborated to systematically integrate IPE simulations into the curricula so that every graduate from the respective schools received TeamSTEPPS® education and participated in a standardized IPE simulation experience
66	Medical student perceptions of an initial collaborative immersion experience	House, J., et al.	This article describes the development of and early outcomes for the initial clinical experience (ICE) course, an innovative collaborative model and a core component of the University of Michigan Medical School’s redesigned curriculum.
67	Decline in medical students’ attitudes to interprofessional learning and patient-centredness	Hudson J., et al	This study explores graduate-entry medical students’ attitudes to IPL and patient-centred care, on programme entry and after an early interdisciplinary clinical experience (ICE).
68	Students’ approaches to learning in clinical interprofessional context	Ponzer, S., et al.	We investigated health care students’ evaluations of interprofessional clinical training in relation to their study orientations.
69	Implementing a nurse-shadowing program for first-year medical students to improve interprofessional collaborations on health care teams	Jain, A., et al.	We then investigated the impact of this intervention on medical students’ knowledge of the roles of nurses as well as their attitudes toward and understanding of the contributions of nurses to the health care team.
70	Examining participant perceptions of an interprofessional simulation-based trauma team training for medical and nursing students	Jakobsen, R., et al.	The aim of this paper is to describe the adaptation of an interprofessional simulation course in an undergraduate setting and to report participants’ experiences with the course and students’ learning outcomes.
71	First Contact: interprofessional education based on medical students’ experiences from their nursing internship	Eich-Krohm, A., et al.	To meet the demographic challenges ahead it is important to emphasize inter- professional education in the study of medicine and better prepare future physicians for interprofessional collaboration.
72	Interprofessional education using simulation of an overnight inpatient ward shift	M. Joyal, K., et al.	The purpose of this study was to investigate the interprofessional knowledge, skills and attitudes the students learn from this experience.
73	A Human Factors Curriculum for Surgical Clerkship Students	Cahan, M., et al.	Early introduction of a full-day human factors training experience into the surgical clerkship curriculum will teach effective communication skills and strategies to gain professional satisfaction from a career in surgery.

**Fig. 3:**
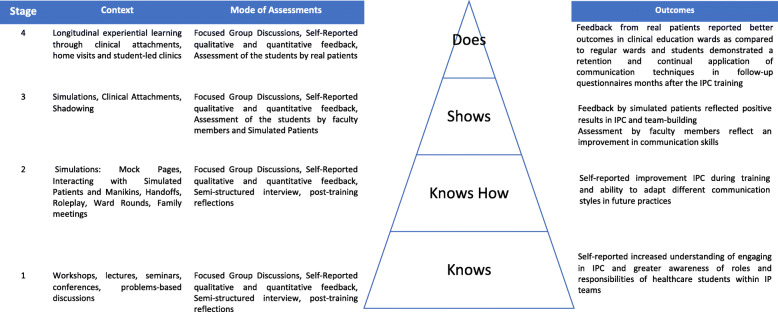
Miller’s Pyramid of Included Articles

Whilst there were accounts that assessed a specific aspect of the IPC process or involved ‘snap shots’ of the IPC process and interactions, accounts of IPC that took a longitudinal perspective of IPC did consider the development of IPC along the 4 levels of Miller’s Pyramid (Fig. 3) [6, 8, 38, 39]. As a result, we present the themes/categories related to each level of Miller’s Pyramid.

#### Level 1: knows

##### Training

Forming the base of the pyramid, the “Knows” level of Miller’s Pyramid focuses on the acquisition of theoretical concepts and skills. IPC training at this level Miller’s Pyramid [6, 8, 38, 39] were part of formal programs. This includes the provision videos, lectures and briefings [40–46], online courses, didactic lectures and workshops [40, 47–53], seminars and conferences [44, 54–56] and even a ‘Healthcare Interprofessional Education Day’ where there opportunities to clarify interprofessional roles and markers of proficiency [57]. IPC training at this level also took place as part of observations of interactions between the healthcare team, role modelled in multidisciplinary settings [58–62].

##### Evaluation

Evaluations at this level of Miller’s Pyramid include self-reported surveys which incorporated checklists, open-ended questions and Likert scales that assessed perception of their own knowledge [40, 43, 47, 50, 54, 55, 57, 59, 63–86]. Focused group discussions [59, 87, 88] and semi-structured interviews were also carried out by faculty members to grade students on their ability to demonstrate their knowledge [52, 64, 65, 89–92]. Only the Mayo High Performance Teamwork Scale [49], the Scope of Practice Checklist [64], Readiness for Interprofessional Learning Scale [93], Conceptions of Learning and Knowledge Questionnaire [94] as well as a purpose-designed questionnaire in Jakobsen, Gran [95]‘s study.

#### Level 2: knows how

##### Training

To achieve the “Knows How” level of Miller’s Pyramid, emphasis was placed on problem-based discussions [49, 53, 56, 65–69, 96–102].

##### Evaluation

Students were asked to reflect on their IPC experiences [45, 56, 62, 72, 103]. In Robertson, Kaplan [104]‘s program, they were asked to point out positive IPC skills demonstrated in a video and suggest areas for improvement.

#### Level 3: shows how

##### Training

The third level of Miller’s pyramid comprises of “Shows How”, where students are required to demonstrate the application of knowledge in their clinical performance. Clinical scenarios included cardiac resuscitations [52, 65, 81, 102]; handoffs [105]; mock pages [41, 106]; communication with a senior clinician [107]; interactions with simulated patients [40, 44, 50, 92, 105] and manikins [46, 49, 73, 74, 76, 95, 108, 109]; simulated ward rounds [43, 45, 48, 51, 71, 110], family meetings [47], roleplay [100, 104]; paediatric clinical simulations [75]; Objective Structured Clinical Exam simulations [111] and laboratory sessions [101]. Non-clinical scenarios incorporated the handling of difficult family conflicts [92] and sensitive cultural issues [92].

##### Evaluation

Student evaluations were carried out by faculty members [65, 69, 77, 99, 105, 108], and supplemented by feedback from simulated patients [49, 97, 99], and team building exercises [97]. A post-training analysis of verbal units of exchange during handoffs also served to quantify improvements in communication skills [105]. Once again only the Mayo High Performance Teamwork Scale [49], the University of West England Interprofessional Questionnaire [89], the Readiness for Interprofessional Learning Scale [65] and the Interprofessional Collaborative Competency Attainment Survey [57] and the I-PASS: Medical Student Workshop [105] were validated.

#### Level 4: does

##### Training

At the apex of the pyramid, the “Does” level focuses on the students’ independent performance in real clinical settings. IPC training was facilitated via experiential learning in clinics [53, 70, 80, 83, 93, 103, 112] and wards [80, 83, 85, 93], student-led clinics [82, 88–90], motivational interviews with certified health educationalists [99] and home visits [78, 91, 113]. These interactions provide opportunities for students to share disciplinary insights and expertise, conduct collaborative medical interviews, explore complex patient cases and manage challenging situations as a unified group [78, 91].

##### Evaluation

Whilst students provided self-reports of their competency levels in clinical settings via questionnaires [79, 89, 96], faculty members [99] and patients [60, 70] were also involved in the observation and identification of good communication skills. Few tools were validated [89, 99] such as the University of West England Interprofessional Questionnaire [89], and the Indiana University Individual Communication Rubric and Indiana University Team Communication Rubric which were modifications of the validated Indiana University Simulation Integration Rubric [99].

##### Attitudes

Acknowledging that IPC experiences and professional and personal development change individual concepts [40, 42, 49, 64, 69, 71, 106, 108], a combination of validated and unvalidated questionnaires, checklists, interviews and reflective pieces were employed to determine prevailing attitudes towards IPC [44, 47, 50, 51, 53, 55, 58, 59, 61, 66–68, 73–77, 80, 82–84, 86–89, 91, 93, 95, 98, 101, 102, 104, 109, 112].

#### Suitability of teaching and evaluation methods

It is of note that across the 73 included studies, only 14 studies [46, 52, 62, 72, 78, 80, 85, 89, 97, 99, 101, 102, 108, 109, 111, 114] offered evaluation methods that appropriately evaluated learning outcomes in a stepwise approach as delineated by the stage(s) of Miller’s pyramid.

Thirty four studies, studied improvements in attitudes towards IPC or satisfaction with training in lieu of assessing any stage in Miller’s pyramid of competency, or, had conducted no assessment [42, 44, 47, 48, 50, 53–55, 58, 59, 61, 67, 68, 71, 73–76, 81–83, 86–88, 90–92, 98, 100, 101, 106, 110, 112, 113].

#### Content of IPC programs

Table 3 describes the list of topics covered in IPC programs. Most interventions were centred around clinical scenarios in various settings, deliberation of ethical issues and care determinations.

**Table 3:** List of topics for IPC Programmes and methods of training

Topics	Studies
**Workshops and Discussion**	
Case learning on medical safety	[55, 96]
Reflecting on videos where nurse and senior resident had different degrees of anxiety about a postoperative patient	[56]
Respiratory Distress	[97]
Antimicrobial Stewardship	[67]
Ethical Case Discussions	[66, 69]
Helping patient’s sister make a decision about goals of care for patient	[47]
**Interviews**	
Interview a nurse and a physician about their experiences with IPC	[62]
**Simulations where interprofessional teams interact such as with each other in the context of a case or a particular scenario and with the aid of manikins and simulated patients**	
Infection Control	[101]
Manikin: Anaphylaxis Chronic obstructive pulmonary disease (COPD), Early sepsis, Acute coronary syndrome and Acute stroke	[109]
Manikin: Respiratory Distress	[81]
Manikin: Cardiac Resuscitation	[102]
Manikin: Management of emergency situations	[45]
Manikin: Paediatric Simulation	[75, 76]
Simulated Patient Meeting: Developing care plan for diabetic patient	[41]
Simulated Patient Meeting: standardized patients exhibiting mild dementia, Parkinson’s disease and frailty	[77]
Simulated Patient: inpatient and outpatient scenario that required interaction of at least three different health professionals. Communication challenges involved dealing with cultural issues, a difficult patient, and family conflict.	[92]
Ward Rounds: Designed based on model developed by Nikendei et al.	[48]
Ward Rounds: Simulated emergency department room for patients being admitted to the inpatient internal medicine service	[43]
Ward Rounds: Communicating results of case discussion to patients	[72]
Ward-based Workshops: Paediatric Medication Safety	[75]
Course-based assessment	[65]
**Experiential Learning with real patients**	
Home Visits: Develop holistic view of implications of childhood impairment by visiting a child with disabilities	[91]
Home Visits: Patients with medication related problems	[78]
Home Visits: For management of Chronic Obstructive Pulmonary Disease (COPD)	[113]
Ward Training with wide variety of orthopaedic diagnoses	[85]
Ward Training: Polypharmacy	[70]
Shadowing: Maternity Care	[87]
Shadowing: Various Healthcare Professionals	[58–60, 79]
Shadowing: Nurses	[61]
**Topics**	**Studies**
Workshops and Discussion	[47, 55, 56, 66, 67, 69, 96, 97]
Interviews	[62]
Simulations	
Infection Control	[101]
Manikins	[45, 75, 76, 81, 102, 109]
Simulated Patients	[41, 77, 92]
Ward Rounds	[43, 48, 72]
Ward-based Workshop	[75]
Course-based Assessment	[65]
Experiential Learning with Real Patients	
Home Visits	[78, 91, 113]
Ward Training	[70, 85]
Shadowing	[58–61, 79, 87]

#### Challenges to IPC training

Challenges to IPC training include scheduling conflicts, difficulties in preparing effective and appropriate programs, obstacles in recruiting [108] and training [72] teachers [56, 80] and students [46, 54, 76, 78]. A further issue is failure to vertically integrate IPC training which has been found to reduce teamwork and collaboration and stunt professional identity [93].

Yet perhaps less evident but nonetheless as concerning is the lack of longitudinal assessment of the IPC interactions [48, 57, 65]. Only one account amongst the 74 included studies employed a longitudinal assessment approach [90].

#### Outcomes of IPC training

A lack of longitudinal assessments limit outcome measures to self-reported increases in understanding and appreciation of IPC [40, 55, 63, 64, 70, 72, 73, 84–86, 91, 107, 112], self-perceived improvements in teamwork [49, 97, 99], communication techniques [107] and clinical communication [49, 97, 99], self-reported improvements in IPC competency [87, 107] and the belief that they would better able to adapt to future practice [40, 47, 51, 57, 59, 62, 63, 66, 70–72, 74, 76, 78, 98, 102, 103, 107, 108].

Critically M. Amerongen, Legros [64], Berger, Mahler [54], Bradley, Cooper [65], Erickson, Blackhall [47], Robertson, Kaplan [104] found that efforts to instil IPC did not result in statistically significant improvements in IPC competencies and attitudes [47, 54, 64, 65, 104]. Bradley, Cooper [65] reported that scores for collaboration decreased three to four months post IPC training. Some have sought to attribute these poor results to cognitive overload [42], a ceiling effect [47] and the need for more training [47]. Concurrently initial discomfort [50] with this communication approach could be countered by continued collaborative work [52, 76, 93] with other healthcare professionals [58, 66, 70, 91, 108].

### Stage 5. Consultations with key stakeholders and synthesis of discussion

Consultations with the expert team and local educators, clinicians and researchers well-versed in IPC training revealed was particularly insightful. To begin these discussions following the review of the omitted data identified through deductive category application [14] and the belief that adopting categories based on Miller’s Pyramid required evidencing, underpinned the decision to adopt Krishna’s ‘Split Approach’ [23–26]. This led to the shift from use of Levac et al. (2015) [24]‘s methodology to scoping reviews to adoption first of the split approach and then the integration of a more structured methodology in the form of a SEBA guided approach to SRs following comments by the journal’s anonymous reviewers.

Discussions with the expert teams and local educators, clinicians and researchers also revealed general consensus that the results of this review aligned with prevailing understandings of IPC programs. It was also agreed upon that there is an urgent need for further research on the impact of IPC training on interprofessional collaborations and in the design of comprehensive and longitudinal training and evaluation programs for medical students.

## Discussion

In addressing its research questions, this scoping review revealed diverse approaches, learning objectives, and methods of assessing IPC in medical schools contribute to the poor alignment of training goals and the desire for a step-wise competency framework [6, 8, 39]. Forty five of the included accounts focused on just one level of the Miller’s pyramid, 23 studies focused on two levels whilst 5 studies considered three levels of Miller’s Pyramid. Critically 59 studies employed inappropriate assessments methods to assess the level of the Miller’s Pyramid employed in their program [115].

Whilst we acknowledge that Miller’s Pyramid is by no means the definitive framework to be used in IPC training, it provides a sound, foundational, learner-centric, progressive scaffolding for the effective acquisition and assimilation of IPC knowledge and skills. There is sufficient data to suggest that IPC programs is best ‘spiralled’ – bearing both vertical and horizontal integration within the curriculum. Whilst each stage builds upon prior core topics, knowledge and skills in a vertical manner, they must also work in tandem horizontally with the wider medical school curricula to ensure that students are equipped with other imperative skills which would adequately prepare them for simulations and clinical placements within their IPC training [116, 117]. This would enable the students to see the interwoven nature of specific cognitive and procedural knowledge and skills across settings, allowing for more judicious decision-making and cohesive interprofessional collaborations.

Likewise, training and evaluation methods must be strategically curated and complementary with this stage-wise curriculum. Evaluations must be longitudinal, holistic, multi-sourced and allow for faculty members to quickly identify areas for remediation. Thus competencies must have both fixed elements and personalised components to contend with the individual needs, abilities and contextual considerations. To this end, portfolios are recommended as a suitable learning and evaluation tool to accompany students as they hone their IPC skills [118–120]. Extensive follow-ups assessing attitudinal and behaviour change [121, 122] should also be conducted following graduation to determine the overall impact of the curriculum on IPC skills into the clinical setting [107].

### Limitations

While it is reassuring that Millers’ Pyramid may be used to address present gaps in IPC training, there are a number of limitations to be broached.

First, drawing from a small pool of papers which were limited to articles published or translated to the English language can be problematic particularly when most are North American and European-centric. This may limit the applicability of the findings in wider healthcare settings.

Two, there is much to be clarified about the IPC training and assessment processes. This endeavor is set back, however, by a lack of holistic and longitudinal assessments and the continued reliance upon assessment tools still rooted in “*Cartesian reductionism and Newtonian principles of linearity*” [123] and fail to consider the evolving nature of the IPC training process and training environment [49, 57, 65].

Three, despite our independent efforts to carry out our searches and independent efforts to verify our searches and consolidate our findings there may still be important articles that have been omitted.

## Conclusion

This scoping review finds that despite efforts to design IPC programs around competency-based stages, most programs lack a longitudinal perspective and effective means of appraising competency. Yet it is still possible to forward a basic framework for the design of IPC programs.

Acknowledging the need for a longitudinal perspective IPC training should be structured around a ‘spiralled’ curriculum. This facilitates both vertical and horizontal integrations within the formal medical training curriculum. Being part of the formal curriculum will also cement IPC as part of the core training processes in medical school and facilitates the recruitment and training of trainers, established purpose built training slots over the course of medical training program, financial support and effective oversight of the program and the training environment. With more medical schools adopting a portfolio-based assessment process, IPC would be furnished with a clear means of longitudinal assessments of IPC competencies over the course of each competency-based stage. It also allows effective follow up of graduates and a link with postgraduate training processes and portfolios.

The program itself must involve all 4 levels of Miller’s Pyramid [6, 8, 38, 39]. For Level 1 of Miller’s Pyramid, a combination of interactive workshops and role modelling of effective IPC in the clinical setting will help medical students appreciate the role of IPC.

Level 2 should involve case based discussions on ethical and care issues in the interprofessional setting whilst Level 3 and 4 may be demonstrated in simulated clinics and ward rounds. Perhaps just as critical is that IPC practice should be regularly assessed in all clinical postings to ensure that remediation can be carried out early.

Being part of the formal curriculum will also ensure that there are quality appraisals of the IPC program and policing of codes of conduct and practice standards. It will also facilitate research into better assessment measures and tools, communication dynamics and the professional identity formation. Finally, it will also evaluate the translatability of these findings beyond medical schools and their links to postgraduate practice.

## Supplementary information

**Additional file 1.** PubMed Search Strategy.

**Additional file 2.** Summary of Included Articles.

**Additional file 3.** PRISMA Checklist.

## Data Availability

All data generated or analysed during this study are included in this published article under Table 2 Indications for an IPC Programme.

## Abbreviations

IPC: Interprofessional collaboration
IPE: Interprofessional education
MDT: Multi-Disciplinary Team
PRISMA: Preferred Reporting Items for Systematic Reviews and Meta-Analyses
