# Supplementary material for: Interprofessional communication (IPC) for medical students: a scoping review
Source: BMC Med Educ. 2020 Oct 16;20:372. doi: 10.1186/s12909-020-02296-x (PMC7574565; doi:10.1186/s12909-020-02296-x)
Supplement: Supplementary file 1 — Additional file 1. PubMed Search Strategy. [file 12909_2020_2296_MOESM1_ESM.docx]

**Supplementary File 1: PubMed Search Strategy**

#1:Students, Medical [Mesh] OR Physicians [Mesh] OR doctor[tiab] OR doctors[tiab] OR physician[tiab] OR physicians[tiab] OR Medical Student [tiab] OR Medical Students [tiab]

#2:((“Students, Nursing”[Mesh] OR Students, Pharmacy [Mesh] OR ((“Students”[Mesh] OR student[tiab] OR students[tiab]) AND ("Social Work Department, Hospital"[Mesh] OR "Physical Therapists"[Mesh] OR "Occupational Therapists"[Mesh] OR "Nurses"[Mesh] OR "Nursing Staff"[Mesh] OR "Physicians"[Mesh] OR Pharmacist [Mesh] OR nurse[tiab] OR nurses[tiab] OR nursing[tiab] OR physiotherapist[tiab] OR physiotherapists[tiab] OR Pharmacist [tiab] OR Pharmacists [tiab] OR "occupational therapist"[tiab] OR "occupational therapists"[tiab] OR "allied health”[tiab] OR (("social worker"[tiab] OR "social workers"[tiab]) AND (clinical[tiab] OR medical[tiab] OR hospital[tiab]))))) OR ("Social Work Department, Hospital"[Mesh] OR "Physical Therapists"[Mesh] OR "Occupational Therapists"[Mesh] Pharmacist [Mesh] OR "Nurses"[Mesh] OR "Nursing Staff"[Mesh] OR nurse[tiab] OR nurses[tiab] OR nursing[tiab] OR physiotherapist[tiab] OR Pharmacist [tiab] OR Pharmacists [tiab] OR physiotherapists[tiab] OR "occupational therapist"[tiab] OR "occupational therapists"[tiab] OR "allied health”[tiab] OR (("social worker"[tiab] OR "social workers"[tiab]) AND (clinical[tiab] OR medical[tiab] OR hospital[tiab]))))

#3:“Interprofessional Relations”[Mesh] OR interprofessional[tiab] OR inter-professional[tiab] OR Interdisciplinary[tiab] OR Inter-disciplinary[tiab] OR Multidisciplinary[tiab] OR multi-disciplinary[tiab] OR Cross-Disciplinary[tiab] OR “physician-nurse”[tiab] OR transprofessional [tiab] OR trans-professional [tiab]

#4: #2 OR #3

#5:((“Communication”[Mesh] OR Communication[tiab] OR Communications[tiab] OR Communicating[tiab] OR Communicate[tiab]) AND (“Delivery of Health Care”[Mesh] OR health[tiab] OR healthcare[tiab] OR medical[tiab] OR clinical[tiab] OR medicine[tiab]) AND (“Education”[Mesh] OR educate[tiab] OR education[tiab] OR educating[tiab] OR educations[tiab] OR curricula[tiab] OR curriculum[tiab] OR teaching[tiab] OR teachings[tiab] OR teach[tiab] OR learn[tiab] OR learning[tiab] OR competence[tiab] OR competency[tiab] OR competencies[tiab]))

#6: #1 AND #4 AND #5
