# Supplementary material for: Interprofessional communication (IPC) for medical students: a scoping review
Source: BMC Med Educ. 2020 Oct 16;20:372. doi: 10.1186/s12909-020-02296-x (PMC7574565; doi:10.1186/s12909-020-02296-x)
Supplement: Supplementary file 2 — Additional file 2. Summary of Included Articles. [file 12909_2020_2296_MOESM2_ESM.docx]

|  |  |  |  | **Pedagogy** | | | | **No. of Stages** | **Assessment** | | | | **Match?** | **Assess Attitudes/Satisfaction ONLY or no assessment** |
| --- | --- | --- | --- | --- | --- | --- | --- | --- | --- | --- | --- | --- | --- | --- |
| **S/N** | **Title** | **Author** | **Millers’ Pyramid Stage** | **Knows** | **Knows How** | **Shows How** | **Does** |  | **Knows** | **Knows How** | **Shows How** | **Does** |  |  |
| 1 | Promoting Interprofessional Collaborative Practice Through Simulation | Alfes, C., et al | Pedagogy:  Shows How, Assessment: Knows, Attitudes and Satisfaction/Feedback with regards to Training |  |  | **/** |  | **1** | **/** |  |  |  | No | No |
| 2 | Design of a successful introductory interprofessional education experience | Helen, M. et al | Pedagogy: Knows and Knows How, Assessment: Knows, Perception of Knowledge and Attitudes and Satisfaction/Feedback with regards to Training | **/** | **/** |  |  | **2** | **/** |  |  |  | No | No |
| 3 | Developing Teamwork Using a Two-Tiered Debriefing Approach Clinical Simulation in Nursing | Andersen, P., et al | Pedagogy: Shows How, Assessment: Attitudes and Satisfaction/Feedback with regards to Training |  |  | **/** |  | **1** |  |  |  |  | No | Yes |
| 4 | Interprofessional learning on polypharmacy | Anderson, E., et al | Pedagogy: Knows How, Assessment: Knows, Perception of Knowledge, Attitudes and Satisfaction/Feedback with regards to Training |  | **/** |  |  | **1** | **/** |  |  |  | No | No |
| 5 | Attitudes Toward Communication and Collaboration After Participation in a Mock Page Program: A Pilot of an Interprofessional Approach to Surgical Residency Preparation | J. Arumpanayil, A., et al | Pedagogy: Shows How, Assessment: Attitudes |  |  | **/** |  | **1** |  |  |  |  | No | Yes |
| 6 | Technology-enabled interprofessional education for nursing and medical students: A pilot study | Berg, B., et al. | Pedagogy: Shows How, Assessment: Shows How and Attitudes and Satisfaction/Feedback with regards to Training |  |  | **/** |  | **1** |  |  | **/** |  | Yes | No |
| 7 | Evaluation of interprofessional education: lessons learned through the development and implementation of an interprofessional seminar on team communication for undergraduate health care students in Heidelberg - a project report | Berger, S., et al. | Pedagogy: Knows How, Shows How, Assessment: Attitudes and Satisfaction/Feedback with regards to Training |  | **/** | **/** |  | **2** |  |  |  |  | No | Yes |
| 8 | Mock pages are a valid construct for assessment of clinical decision making and interprofessional communication | Boehler, M. L., et al. | Pedagogy: Knows, Knows How, Shows How, Assessment: Shows How | **/** | **/** | **/** |  | **3** |  |  | **/** |  | No | No |
| 9 | A mixed-methods study of interprofessional learning of resuscitation skills | Bradley, P., et al. | Pedagogy: Knows, Shows How, Assessment: Shows How | **/** |  | **/** |  | **2** |  |  | **/** |  | No | No |
| 10 | Examining the effects of interprofessional problem-based clinical ethics: Findings from a mixed methods study | Chihchen Chou, F., et al. | Pedagogy: Knows, Knows How, Assessment: Shows How, Attitudes and Satisfaction/Feedback with regards to Training | **/** | **/** |  |  | **2** |  |  | **/** |  | No | No |
| 11 | The role of a multidisciplinary student team in the community management of chronic obstructive pulmonary disease | Clarke, H. and M. Voss | Pedagogy: Does, Assessment: NA |  |  |  | **/** | **1** |  |  |  |  | No | Yes |
| 12 | Fostering interprofessional communication through case discussions and simulated ward rounds in nursing and medical education: A pilot project | Wershofen, B., et al. | Pedagogy: Knows How, Shows How, Assessment: Knows How, Shows How |  | **/** | **/** |  | **2** |  | **/** | **/** |  | Yes | No |
| 13 | Learning to Overcome Hierarchical Pressures to Achieve Safer Patient Care: An Interprofessional Simulation for Nursing, Medical, and Physician Assistant Students | Reeves, S. A., et al. | Pedagogy: Knows, Shows How, Assessment: Does, Satisfaction/Feedback with regards to Training | **/** |  | **/** |  | **2** |  |  |  | **/** | No | No |
| 14 | An inter-professional approach to train and evaluate communication accuracy and completeness during the delivery of nurse-physician student handoffs | Maraccini, A., et al. | Pedagogy: Knows, Shows How, Assessment: Shows How | **/** |  | **/** |  | **2** |  |  | **/** |  | No | No |
| 15 | Interprofessional simulation-based education program: a promising approach for changing stereotypes and improving attitudes toward nurse-physician collaboration | Liaw, S. Y., et al. | Pedagogy: Shows How, Assessment: Attitudes |  |  | **/** |  | **1** |  |  |  |  | No | Yes |
| 16 | Teamwork training with nursing and medical students: Does the method matter? Results of an interinstitutional, interdisciplinary collaboration | Hobgood, C., et al. | Pedagogy: Knows, Knows How, Shows How, Assessment: Knows, Shows How, Attitudes | **/** | **/** | **/** |  | **3** | **/** |  | **/** |  | No | No |
| 17 | An Interprofessional Workshop for Students to Improve Communication and Collaboration Skills in End-of-life Care | Erickson, J. M., et al. | Pedagogy: Knows How, Shows How, Assessment: Attitudes |  | **/** | **/** |  | **2** |  |  |  |  | No | Yes |
| 18 | COMPARISON of Communication Outcomes in Traditional VERSUS Simulation Strategies in Nursing and Medical Students | Reising, D. L., et al. | Pedagogy: Shows How, Assessment: Attitudes |  |  | **/** |  | **1** |  |  |  |  | No | Yes |
| 19 | The use of simulation and a modified TeamSTEPPS curriculum for medical and nursing student team training simulation | Robertson, B., et al. | Pedagogy: Knows, Knows How, Shows How, Assessment: Knows How, Attitudes and Satisfaction/Feedback with regards to Training | **/** | **/** | **/** |  | **3** |  | **/** |  |  | No | No |
| 20 | "Collaborative-ready" students: Exploring factors that influence collaboration during a longitudinal interprofessional education practice experience | Rotz, M. and G. Dueñas | Pedagogy: Shows How, Assessment: NA |  |  | **/** |  | **1** |  |  |  |  | No | Yes |
| 21 | Medical school hotline: interprofessional education: future nurses and physicians learning together | Sakai, D. H., et al. | Pedagogy: Shows How, Assessment: NA |  |  | **/** |  | **1** |  |  |  |  | No | Yes |
| 22 | A novel interprofessional shadowing initiative for senior medical students | Shafran, D. M., et al. | Pedagogy: Knows, Assessment: Attitudes and Satisfaction/Feedback with regards to Training | **/** |  |  |  | **1** |  |  |  |  | No | Yes |
| 23 | The student-run free clinic: An ideal site to teach interprofessional education? | Sick, B., et al. | Pedagogy: Shows How, Does, Assessment: Attitudes, Does and Shows How, Perception of Competency |  |  | **/** | **/** | **2** |  |  | **/** | **/** | Yes | No |
| 24 | Undergraduate students' perceptions of and attitudes toward a simulation-based interprofessional curriculum: the KidSIM ATTITUDES questionnaire | Sigalet, E., et al. | Pedagogy: Shows How, Assessment: Attitudes |  |  | **/** |  | **1** |  |  |  |  | No | Yes |
| 25 | Evaluation of an Interprofessional Education Communication Skills Initiative | Solomon, P. and J. Salfi | Pedagogy: Shows How, Assessment: Attitudes and Satisfaction/Feedback with regards to Training |  |  | **/** |  | **1** |  |  |  |  | No | Yes |
| 26 | Undergraduate interprofessional education using high-fidelity paediatric simulation | Stewart, M., et al. | Pedagogy: Shows How, Assessment: Attitudes, Satisfaction/Feedback with regards to Training |  |  | **/** |  | **1** |  |  |  |  | No | Yes |
| 27 | An interprofessional approach to improving paediatric medication safety | Stewart, M., et al. | Pedagogy: Shows How, Assessment: Attitudes, Satisfaction/Feedback with regards to Training |  |  | **/** |  | **1** |  |  |  |  | No | Yes |
| 28 | Child disability case studies: an interprofessional learning opportunity for medical students and paediatric nursing students | Street, K. N., et al. | Pedagogy: Knows How, Shows How, Assessment: Attitudes, Satisfaction/Feedback with regards to Training |  | **/** | **/** |  | **2** |  |  |  |  | No | Yes |
| 29 | Interprofessional simulation training improves knowledge and teamwork in nursing and medical students during internal medicine clerkship | M Tofil, N., et al. | Pedagogy: Shows How, Assessment: Knows, Perception of Knowledge, Satisfaction/Feedback with regards to Training |  |  | **/** |  | **1** | **/** |  |  |  | No | No |
| 30 | Interprofessional training enhances collaboration between nursing and medical students: A pilot study | Turrentine, B., et al. | Pedagogy: Knows, Shows How, Assessment: Attitudes, Shows How, Perception of Competency | **/** | **/** |  |  | **2** |  |  | **/** |  | No | No |
| 31 | Analysis of an interprofessional home visit assignment: student perceptions of team-based care, home visits, and medication-related problems | Vaughn, L. M., et al. | Pedagogy: Shows How, Assessment: Shows How, Perception of Competency |  |  | **/** |  | **1** |  |  | **/** |  | Yes | No |
| 32 | Developing interprofessional communication skills | Wagner, J., et al. | Pedagogy: Shows How, Assessment: NA |  |  | **/** |  | **1** |  |  |  |  | No | Yes |
| 33 | Reflections and unprompted observations by healthcare students of an interprofessional shadowing visit | Wright, A., et al. | Pedagogy: Knows, Knows How, Assessment: Attitudes | **/** | **/** |  |  | **2** |  |  |  |  | No | Yes |
| 34 | Interprofessional education: The student perspective | Lumague, M., et al. | Pedagogy: Knows, Knows How, Assessment: Attitudes, Satisfaction/Feedback with regards to Training | **/** | **/** |  |  | **2** |  |  |  |  | No | Yes |
| 35 | An introductory interprofessional exercise for healthcare students | Macdonnell, C., et al. | Pedagogy: Shows How, Assessment: Shows How |  |  | **/** |  | **1** |  |  | **/** |  | Yes | No |
| 36 | An Interprofessional Curriculum on Antimicrobial Stewardship Improves Knowledge and Attitudes Toward Appropriate Antimicrobial Use and Collaboration | MacDougall, C., et al. | Pedagogy: Shows How, Assessment: Attitudes |  |  | **/** |  | **1** |  |  |  |  | No | Yes |
| 37 | An interprofessional education pilot program in maternity care: Findings from an exploratory case study of undergraduate students | Meffe, F., et al. | Pedagogy: Knows, Knows How, Shows How, Assessment: Attitudes | **/** | **/** | **/** |  | **3** |  |  |  |  | No | Yes |
| 38 | Innovation in learning - An inter-professional approach to improving communication | Mitchell, M., et al. | Pedagogy: Knows, Knows How, Assessment: Attitudes, Satisfaction/Feedback with regards to Training | **/** | **/** |  |  | **2** |  |  |  |  | No | Yes |
| 39 | Sustained effects of interprofessional shared learning on student attitudes to communication and team working depend on shared learning opportunities on clinical placement as well as in the classroom | Morison, S. and J. Jenkins | Pedagogy: Shows How, Assessment: Attitudes |  |  | **/** |  | **1** |  |  |  |  | No | Yes |
| 40 | An interprofessional communication skills lab: A pilot project | Salvatori, P., et al. | Pedagogy: Shows How, Assessment: Satisfaction/Feedback with regards to Training |  |  | **/** |  | **1** |  |  |  |  | No | Yes |
| 41 | Evaluation of a Unique Interprofessional Education Program Involving Medical and Pharmacy Students | J. Nagge, J., et al. | Pedagogy: Knows, Shows How, Assessment: Shows How, Perception of Competency | **/** |  | **/** |  | **2** |  |  | **/** |  | No | No |
| 42 | Integrating Collaborative Interprofessional Simulation into Pre-Licensure Health Care Programs | New, S. N., et al. | Pedagogy: Knows, Shows How, Assessment: Attitudes, Satisfaction/Feedback with regards to Training | **/** |  | **/** |  | **2** |  |  |  |  | No | Yes |
| 43 | Students' Perceptions on an Interprofessional Ward Round Training - A Qualitative Pilot Study | Nikendei, C., et al. | Pedagogy: Shows How, Assessment: Attitudes |  |  | **/** |  | **1** |  |  |  |  | No | Yes |
| 44 | How can student experience enhance the development of a model of interprofessional clinical skills education in the practice placement setting? | O'Carroll, V., et al. | Pedagogy: Knows How, Assessment: Satisfaction/Feedback with regards to Training |  | **/** |  |  | **1** |  |  |  |  | No | Yes |
| 45 | Students' understanding of teamwork and professional roles after interprofessional simulation-a qualitative analysis | Oxelmark, L., et al. | Pedagogy: Knows, Knows How, Assessment: Knows How, Satisfaction/Feedback with regards to Training | **/** | **/** |  |  | **2** |  | **/** |  |  | No | No |
| 46 | Medical students' engagement in collaborative communication during an interprofessional standardized patient encounter | K. Oza, S., et al. | Pedagogy: Shows How, Assessment: Shows How |  |  | **/** |  | **1** |  |  | **/** |  | Yes | No |
| 47 | Development and implementation of an interprofessional pharmacotherapy learning experience during an advanced pharmacy practice rotation in primary care | Patel, K., et al. | Pedagogy: Knows How, Assessment: Attitudes |  | **/** |  |  | **1** |  |  |  |  | No | Yes |
| 48 | A mile in their shoes: interdisciplinary education at the Johns Hopkins University School of Medicine | Pathak, S., et al. | Pedagogy: Knows, Assessment: Knows, Does, Perception of Knowledge and Competency | **/** |  |  |  | **1** | **/** |  |  | **/** | No | No |
| 49 | Interprofessional training in the context of clinical practice: goals and students' perceptions on clinical education wards | Ponzer, S., et al. | Pedagogy: Knows, Shows How, Assessment: Attitudes: Knows, Shows How, Perception of Knowledge and Competency | **/** |  | **/** |  | **2** | **/** |  | **/** |  | Yes | No |
| 50 | Evaluating an undergraduate interprofessional simulation-based educational module: communication, teamwork, and confidence performing cardiac resuscitation skills | Luctkar-Flude, M., et al. | Pedagogy: Knows, Assessment: Attitudes, Satisfaction/Feedback with regards to Training | **/** |  |  |  | **1** |  |  |  |  | No | Yes |
| 51 | Improving collaboration among medical, nursing and respiratory therapy students through interprofessional simulation | Elizabeth Ann King, A., et al. | Pedagogy: Shows How, Assessment: Satisfaction/Feedback with regards to Training |  |  | **/** |  | **1** |  |  |  |  | No | Yes |
| 52 | Interprofessional education for the quality use of medicines: Designing authentic multimedia learning resources | Levett-Jones, T., et al. | Pedagogy: Knows, Assessment: Knows, Knows How, Shows How/Does | **/** |  |  |  | **1** | **/** | **/** | **/** | **/** | No | No |
| 53 | What and how do students learn in an interprofessional student-run clinic? An educational framework for team-based care | Lie, D., et al. | Pedagogy: Shows How, Assessment: Attitudes, Satisfaction/Feedback with regards to Training |  |  | **/** |  | **1** |  |  |  |  | No | Yes |
| 54 | The impact of an interprofessional problem-based learning curriculum of clinical ethics on medical and nursing students' attitudes and ability of interprofessional collaboration: A pilot study | Lin, Y.-C., et al. | Pedagogy: Knows, Knows How, Assessment: Attitudes, Knows, Perception of Knowledge | **/** | **/** |  |  | **2** | **/** |  |  |  | No | No |
| 55 | Interprofessional learning through shadowing: Insights and lessons learned | V. Kusnoor, A. and L. A. Stelljes | Pedagogy: Knows, Shows How, Assessment: Knows How | **/** |  | **/** |  | **2** |  | **/** |  |  | Nov | No |
| 56 | Interprofessional student-led clinics: An innovative approach to the support of older people in the community | Kent, F., et al. | Pedagogy: Shows How, Assessment: Attitudes, Satisfaction/Feedback with regards to Training |  |  | **/** |  | **1** |  |  |  |  | No | Yes |
| 57 | Interprofessional clinical training for undergraduate students in an emergency department setting | Ericson, A., et al. | Pedagogy: Knows How, Shows How, Assessment: Attitudes, Satisfaction/Feedback with regards to Training |  | **/** | **/** |  | **2** |  |  |  |  | No | Yes |
| 58 | Indonesian students' participation in an interprofessional learning workshop | Ernawati, D., et al. | Pedagogy: Knows How, Assessment: Attitudes |  | **/** |  |  | **1** |  |  |  |  | No | Yes |
| 59 | Nursing and medical students teaming up: Results of an interprofessional project | Feather, R., et al. | Pedagogy: Does, Assessment: Does |  |  |  | **/** | **1** |  |  |  | **/** | Yes | No |
| 60 | Simulating a patient's fall as a means to improve routine communication: Joint training for nursing and fifth-year medical students | Flentje, M., et al. | Pedagogy: Shows How, Assessment: Shows How, Satisfaction/Feedback with regards to Training |  |  | **/** |  | **1** |  |  | **/** |  | Yes | No |
| 61 | Effects of interprofessional education on patient perceived quality of care | Hallin, K., et al. | Pedagogy: Does, Assessment: Does |  |  |  | **/** | **1** |  |  |  | **/** | Yes | No |
| 62 | Active interprofessional education in a patient based setting increases perceived collaborative and professional competence | Hallin, K., et al. | Pedagogy: Does, Assessment: Knows, Perception of Knowledge, Attitudes |  |  |  | **/** | **1** | **/** |  |  |  | No | No |
| 63 | Interprofessional working in acute care | Holland, C., et al. | Pedagogy: Shows How, Assessment: Attitudes, Shows How, Perception of Competency, Satisfaction/Feedback with regards to Training |  |  | **/** |  | **1** |  |  | **/** |  | Yes | No |
| 64 | Development of an interprofessional educational module on infection control using high-fidelity patient simulation | Luctkar-Flude, M., et al. | Pedagogy: Shows How, Assessment: Attitudes, Shows How, Perception of Competency, Satisfaction/Feedback with regards to Training |  |  | **/** |  | **1** |  |  | **/** |  | Yes | No |
| 65 | Developing a Foundation for Interprofessional Education Within Nursing and Medical Curricula | Leann Horsley, T., et al. | Pedagogy: Shows How, Assessment: Shows How |  |  | **/** |  | **1** |  |  | **/** |  | Yes | No |
| 66 | Medical student perceptions of an initial collaborative immersion experience | House, J., et al. | Pedagogy: Knows How, Does, Assessment: Knows How |  | **/** |  | **/** | **2** |  | **/** |  |  | No | No |
| 67 | Decline in medical students' attitudes to interprofessional learning and patient-centredness | Hudson J., et al | Pedagogy: Knows, Knows How, Does, Assessment: Attitudes, Knows | **/** | **/** |  | **/** | **3** | **/** |  |  |  | No | No |
| 68 | Students' approaches to learning in clinical interprofessional context | Ponzer, S., et al. | Pedagogy: Does, Assessment: Knows, Perception of Knowledge, Attitudes and Satisfaction/Feedback with regards to Training |  |  |  | **/** | **1** | **/** |  |  |  | No | No |
| 69 | Implementing a nurse-shadowing program for first-year medical students to improve interprofessional collaborations on health care teams | Jain, A., et al. | Pedagogy: Knows, Shows How, Assessment: Satisfaction/Feedback with regards to Training, Attitudes | **/** |  | **/** |  | **2** |  |  |  |  | No | Yes |
| 70 | Examining participant perceptions of an interprofessional simulation-based trauma team training for medical and nursing students | Jakobsen, R., et al. | Pedagogy: Shows How, Assessment: Knows, Satisfaction/Feedback with regards to Training, Attitudes |  |  | **/** |  | **1** | **/** |  |  |  | No | No |
| 71 | First Contact: interprofessional education based on medical students' experiences from their nursing internship | Eich-Krohm, A., et al. | Pedagogy: Knows How, Assessment: Knows How |  | **/** |  |  | **1** |  | **/** |  |  | Yes | No |
| 72 | Interprofessional education using simulation of an overnight inpatient ward shift | M. Joyal, K., et al. | Pedagogy: Shows How, Assessment: Attitudes, Knows, Perception of Knowledge |  |  | **/** |  | **1** | **/** |  |  |  | No | No |
| 73 | A Human Factors Curriculum for Surgical Clerkship Students | Cahan, M., et al. | Pedagogy: Knows, Assessment, Knows How | **/** |  |  |  | **1** |  | **/** |  |  | No | No |
